# Refractory paradoxical pustular psoriasis in the setting of synovitis, acne, pustulosis, hyperostosis, osteitis syndrome treated by spesolimab

**DOI:** 10.1016/j.jdcr.2026.07.057

**Published:** 2026-08-04

**Authors:** Iraj Hasan, Andrea Medina-Gonzalez, Jessica Patterson

**Affiliations:** a Department of Medicine, West Virginia University, Morgantown, West Virginia; b Department of Dermatology, West Virginia University, Morgantown, West Virginia

**Keywords:** Crohn’s disease, palmoplantar psoriasis, pustular psoriasis, SAPHO, spesolimab

## Introduction

SAPHO syndrome (synovitis-acne-pustulosis-hyperostosis-osteitis) is a spectrum of rare inflammatory bone, joint, and skin disorders characterized by variable combinations of aseptic neutrophilic dermatoses and aseptic osteoarticular lesions.^1^ Palmoplantar pustulosis is the classic cutaneous manifestation of SAPHO syndrome, although other pustular dermatoses, including pustular psoriasis, have been reported. Generalized pustular psoriasis is characterized by widespread sterile pustules and is increasingly recognized as a distinct inflammatory disorder associated with dysregulated interleukin 36 (IL-36) signaling.^2^ Spesolimab, an IL-36 receptor antagonist, has recently emerged as a promising treatment for pustular psoriasis.^3^ Paradoxical pustular psoriasiform eruptions have been described following TNF-alpha inhibitor treatments for inflammatory bowel disease.^4^ Here, we describe a patient with refractory widespread pustular psoriasis presenting after infliximab treatment in the setting of SAPHO syndrome, with a substantial response to the IL-36 inhibitor spesolimab.

## Case report

A 21-year-old woman with a history of Crohn’s disease, acne, and hidradenitis suppurativa developed an eruption of erythematous, sterile pustules and papules on the back, legs, forearms, hands, and feet in August while receiving infliximab for Crohn’s disease (Fig 1). Histopathology showed psoriasiform epidermal acanthosis with parakeratosis, neutrophilic aggregates in the upper spinous layer and stratum corneum, and spongiform pustulation, consistent with pustular psoriasis. She also reported pain in the chest, back, hips, shoulders, and knees. A nuclear bone scan demonstrated focal intense radiotracer uptake with destructive changes in the sternum, as well as uptake in the sternoclavicular joints and ribs (Fig 2). Taken together, the pustular eruption with both palmoplantar and more widespread psoriasiform features, the polyarthralgia, and the characteristic bone scan findings arising in the context of tumor necrosis factor α (TNF-α) inhibitor therapy supported a unifying diagnosis of drug-induced SAPHO syndrome.

**Fig 1:**
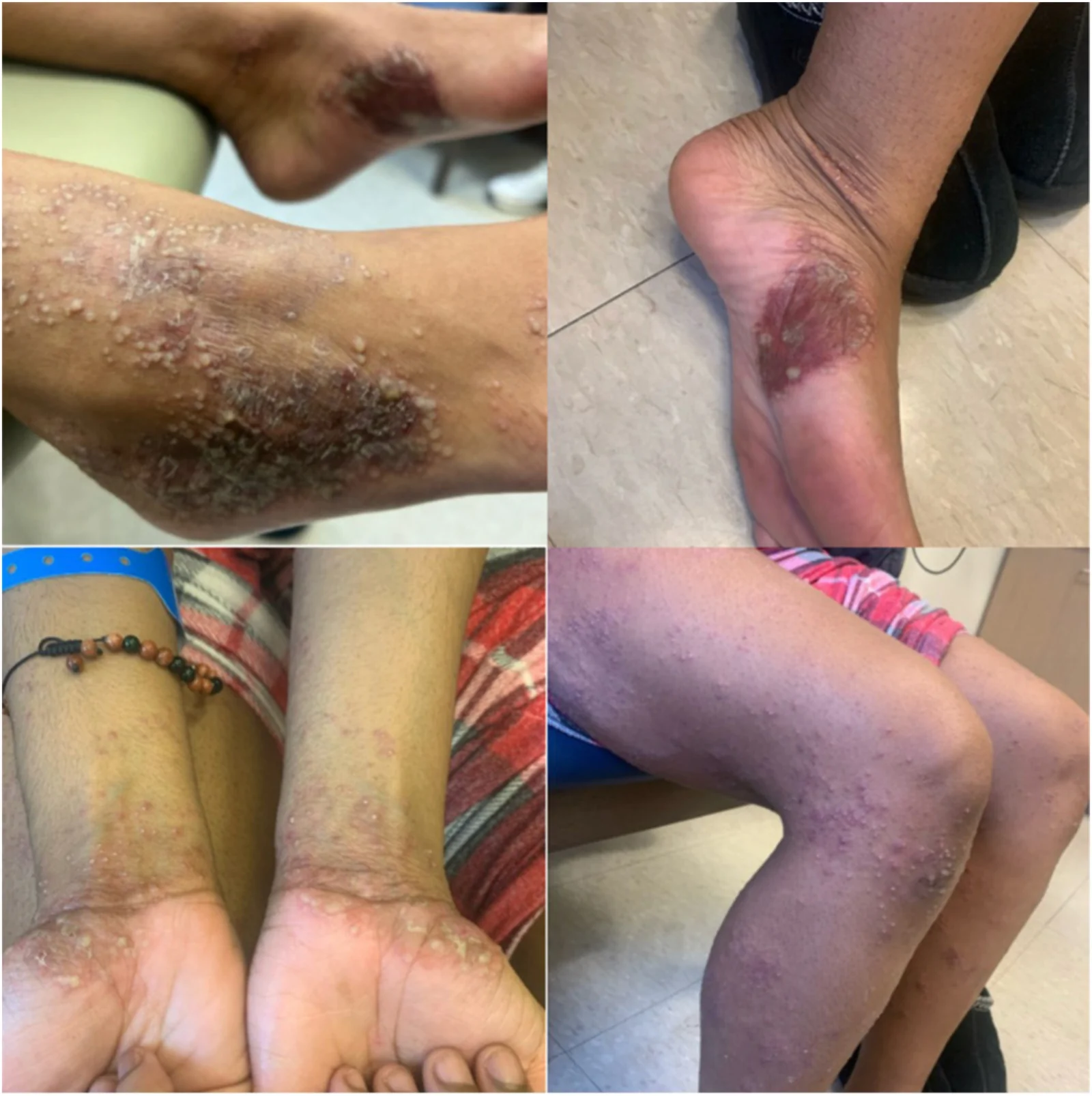
Initial presentation, with erythematous, scaly plaques, and numerous pustules.

**Fig 2:**
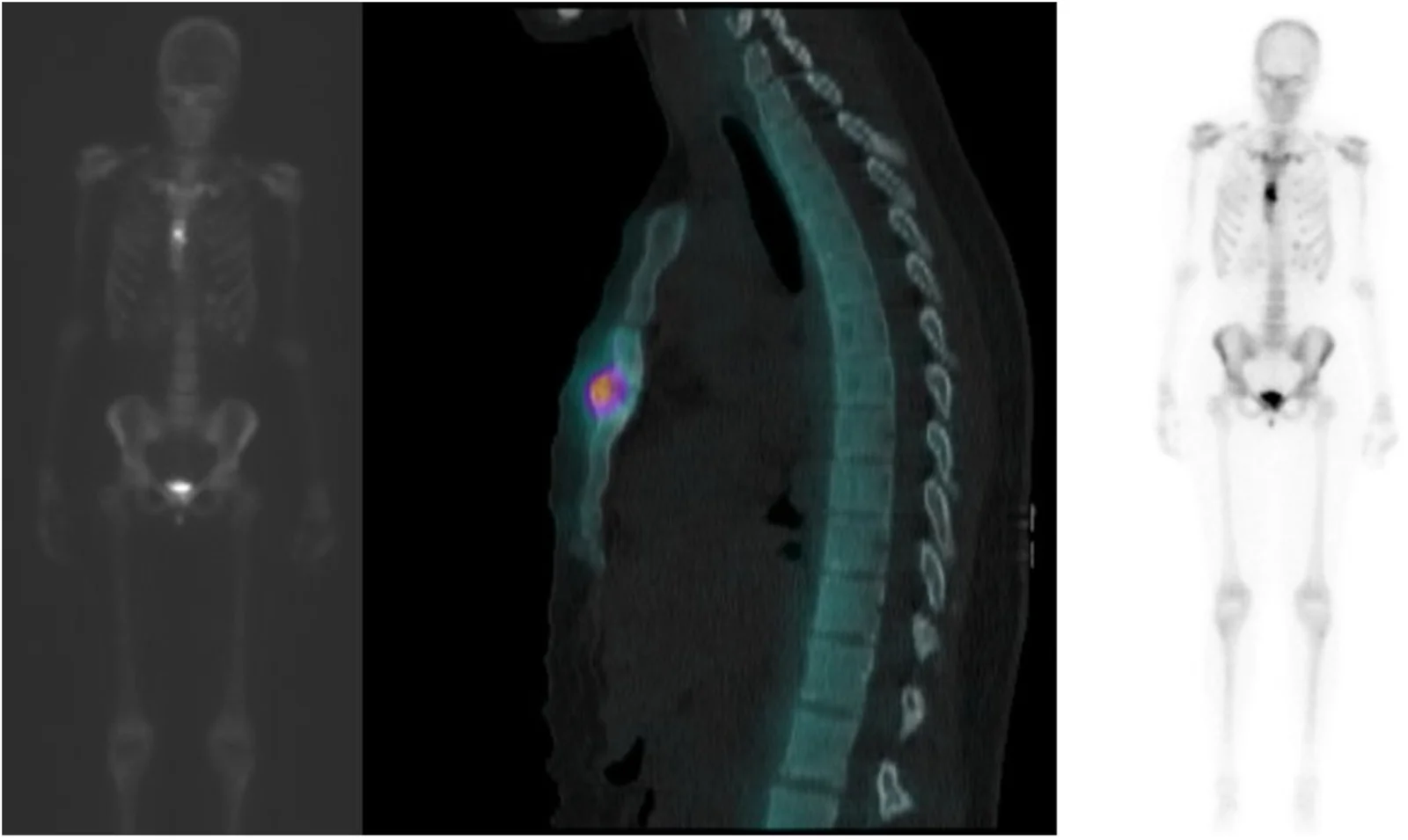
Nuclear bone scan images showing radiotracer uptake in the sternum, sternoclavicular joints, and ribs.

Infliximab was discontinued in October, and her Crohn’s disease was subsequently managed with upadacitinib. The eruption initially improved following removal of the TNF-α trigger, consistent with the partial spontaneous remission that has been described in some cases of drug-induced SAPHO after the offending agent is withdrawn. However, within 1 month the eruption recurred diffusely, beginning at the wrist and spreading to the feet, trunk, and genitals, suggesting that the disease had become self-sustaining independent of the original trigger. Upadacitinib was then discontinued in December given lack of benefit for the skin disease, and she was treated sequentially with cyclosporine and risankizumab without improvement. The eruption progressed with diffuse pustules, scaling, and worsening musculoskeletal pain. No additional Crohn’s-directed therapy was initiated during this period.

In January of the following year, spesolimab 900 mg intravenously was initiated, and other systemic therapies were discontinued. After 2 infusions, the diffuse eruption improved considerably, with residual pustules confined to the hands and feet. Methotrexate 12.5 mg weekly with folic acid was added for Crohn’s disease and additional cutaneous control but was later discontinued due to minimal improvement and concern for hepatotoxicity.

Over the following months, her eruption remained well controlled, with disease limited to acral sites and marked improvement in erythema and desquamation (Fig 3). She has not developed any new pustules and areas healed with postinflammatory hyperpigmentation. Chest and hip pain improved, though intermittent discomfort persisted. At her most recent follow-up, 1 year after initiation of spesolimab, she reported a markedly improved quality of life with minimal skin activity. Her acne and hidradenitis suppurativa also demonstrated significant clinical improvement and remain well controlled on spesolimab. Rheumatology recommended adding hydroxychloroquine for persistent arthritis symptoms. Notably, spesolimab appears to be controlling her Crohn’s disease as a single agent: at her most recent gastroenterology visit she reported 1-3 formed bowel movements per day with no other gastrointestinal symptoms and had regained previously lost weight. Repeat nuclear bone scan has not yet been performed; however, pelvic magnetic resonance imaging has been scheduled to further assess the musculoskeletal response to therapy. This evaluation will be important in determining whether additional disease-modifying treatment targeting the osseous manifestations of SAPHO is warranted.

**Fig 3:**
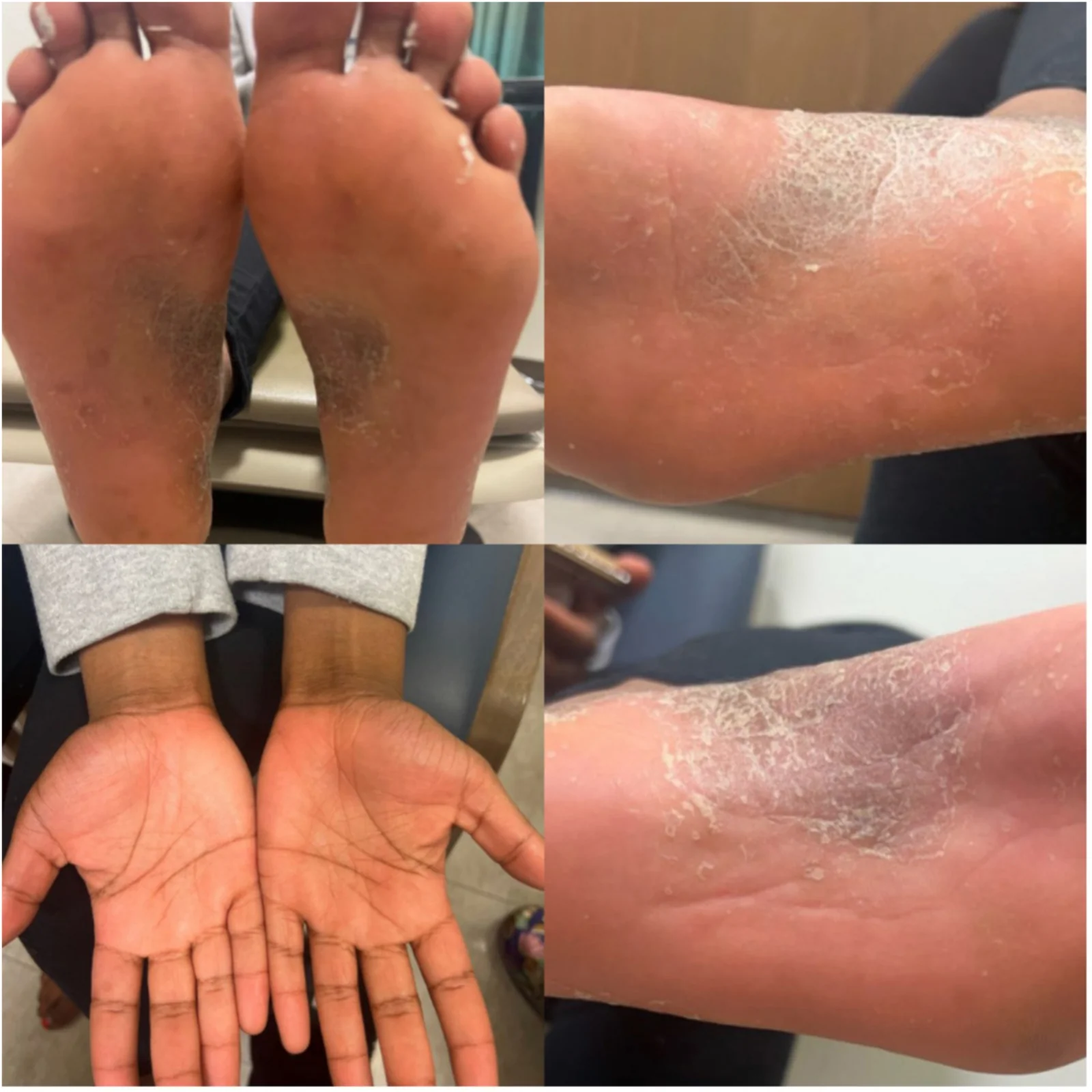
Ten months after initial presentation. Majority of prior pustules and erythema resolved, with residual scaly plaques.

## Discussion

This case illustrates TNF-α inhibitor-induced pustular psoriasis presenting with SAPHO syndrome. Multiple systemic agents, including cyclosporine, risankizumab, and methotrexate, failed to control disease. Spesolimab produced rapid and sustained improvement in cutaneous symptoms, along with reduction in joint pain and continued remission of Crohn’s disease.

Paradoxical pustular psoriasis and SAPHO syndrome have been reported during TNF-α inhibitor therapy, particularly in patients with inflammatory bowel disease.^4^ This case may be best understood as drug-induced SAPHO syndrome, in which the TNF-α inhibitor triggered both the cutaneous pustular eruption and the musculoskeletal disease as manifestations of a single inflammatory entity. The natural history of drug-induced SAPHO is not well characterized; spontaneous remission following removal of the offending drug has been reported in some cases,^5^ but our patient’s partial improvement after infliximab discontinuation followed by subsequent relapse illustrates that persistence and progression can occur despite drug withdrawal, suggesting the inflammatory cascade had become self-sustaining. The marked response to spesolimab raises the question of why some pustular variants respond selectively to IL-36 inhibition; this may reflect disease subtypes with predominant IL-36 pathway dysregulation, a finding that warrants further study.

To the best of our knowledge, this represents the first reported case of spesolimab used successfully for refractory drug-induced SAPHO with pustular psoriasis. Importantly, determining whether spesolimab also improves the radiographic and osseous manifestations of SAPHO syndrome, given the patient's significant extracutaneous disease burden, would be valuable. This information could help clarify whether additional therapies targeting the musculoskeletal component of the disease are necessary. This case emphasizes the need to recognize paradoxical psoriasis in patients on TNF-α inhibitors, to consider a diagnosis of SAPHO in pustular psoriasis with musculoskeletal pain, and highlights IL-36 inhibition as a promising therapy for refractory pustular variants, particularly in the setting of comorbid inflammatory bowel disease.

## Conflicts of interest

None disclosed.
